# Anti-citrullinated peptide autoantibodies, human leukocyte antigen shared epitope and risk of future rheumatoid arthritis: a nested case–control study

**DOI:** 10.1186/ar4342

**Published:** 2013-10-23

**Authors:** Elizabeth V Arkema, Barbara L Goldstein, William Robinson, Jeremy Sokolove, Catriona A Wagner, Susan Malspeis, Bernard Rosner, Francine Grodstein, Elizabeth W Karlson, Karen H Costenbader

**Affiliations:** 1 Division of Rheumatology, Immunology and Allergy, Department of Medicine, Brigham and Women’s Hospital, Harvard Medical School, 75 Francis Street, Boston, MA 02115, USA; 2 Department of Epidemiology, Harvard School of Public Health, 677 Huntington Avenue, Boston, MA 02115, USA; 3 Division of Immunology and Rheumatology, Department of Medicine, Stanford University School of Medicine CCSR, 269 Campus Drive, Stanford, CA 94305, USA; 4 Department of Biostatistics, Harvard School of Public Health, 677 Huntington Avenue, Boston, MA 02115, USA; 5 Channing Division of Network Medicine, Department of Medicine, Brigham and Women’s Hospital, Harvard Medical School, 181 Longwood Avenue, Boston, MA 02115, USA

## Abstract

**Introduction:**

The aim of this study was to characterize anti-citrullinated peptide antibody (ACPA) serostatus in pre-clinical rheumatoid arthritis (RA) with and without Human Leukocyte Antigen-Shared Epitope (*HLA-SE*) alleles.

**Methods:**

We identified 192 women in the Nurses’ Health Study cohorts with blood samples obtained 4 months to 17 years prior to medical record-confirmed RA diagnosis. Three controls were selected matched on age, cohort, menopausal status and post-menopausal hormone use. Reactivities to 18 ACPAs were measured using a custom BioPlex platform. We used conditional logistic regression to calculate the relative risk (RR) of RA for any ACPA-positive and peptide-specific ACPA-positive and examined RRs by time between blood draw and RA onset. Measures of multiplicative and additive interaction between any ACPA-positive and *HLA-SE* were calculated.

**Results:**

All ACPAs by peptide groups were significantly associated with RA risk, RRs ranged from 4.7 to 11.7. The association between ACPA and RA varied over time with the strongest association in those with blood draw less than 5 years before onset (RR 17.0 [95% CI 5.8 to 53.7]) and no association 10 or more years prior to onset (RR 1.4 [95% CI 0.5 to 4.3]). Individuals with both *HLA-SE* and any ACPA-positive had the highest risk of RA. *HLA-SE*-positive RA cases showed reactivity to more ACPA types than *HLA-SE* negative (χ^2^ test for trend, *P* = 0.01).

**Conclusions:**

There is increasing ACPA reactivity up to 10 years before RA onset with the strongest association within 5 years of RA onset. The magnitude of the response to ACPAs, in combination with the presence of *HLA-SE*, is most important for identifying those individuals with the highest risk of RA.

## Introduction

Rheumatoid arthritis (RA) is an autoimmune inflammatory arthropathy characterized by inflammation and autoantibodies directed against citrullinated peptides. The anticyclic citrullinated peptide (anti-CCP) test, a commercial assay that relies upon reactivity to several modified or synthetic CCPs, is a useful assay for diagnosing RA and is highly specific for RA [1]. Recently, research has focused on autoantibodies directed against specific proteins and/or peptides found in the synovium, such as vimentin, fibrinogen and enolase [2-7], in an effort to identify a specific antigen responsible for inciting RA. Patients with RA may be positive for more than one anticitrullinated peptide antibody (ACPA), and there appears to be epitope spreading of ACPA reactivity in the years leading up to diagnosis [5,6,8-10].

Human leukocyte antigen (HLA)*-*DRB1 shared epitope (*HLA-SE*) alleles are the strongest genetic risk factors for RA, carrying a two- to threefold increased risk [11-13]. Past case–control studies have found that having both *HLA-SE* and anti-CCP antibodies confers a greater risk of developing RA than either one alone [14-16]. Among individuals with RA, those with *HLA-SE* alleles are more likely to have anti-CCP antibodies [2,4,17-21].

Most studies of individual ACPAs and RA risk have included patients with early or well-established RA, and those that have included pre-RA patients have not examined the combined effect of ACPA and *HLA-SE*. Our objective in the present study was to characterize reactivity to ACPAs targeted to epitopes found in the rheumatoid synovium in the preclinical period before RA onset using a nested case–control design within the Nurses’ Health Study (NHS) and Nurses’ Health Study II (NHSII) cohorts. We examined the relationship between the number of ACPAs recognized and the time interval until RA symptom onset. Furthermore, we aimed to understand the interaction between ACPA and *HLA-SE* in determining RA risk within this preclinical window.

## Methods

### Study design and population

The NHS is a prospective cohort of 121,700 female nurses ages 30 to 55 years living in 11 states in 1976. The NHSII is a similar cohort started in 1989 of 116,430 female nurses ages 25 to 42 years living in 14 states in the United States. The NHS and NHSII participants completed questionnaires at baseline and every 2 years afterward regarding diseases, lifestyle and health practices. From 1989 to 1990, 32,826 NHS participants (27%) provided blood samples, and from 1996 and 1999, 29,611 NHSII participants (25%) provided blood samples for future studies. We excluded women with any history of cancer (except nonmelanoma skin cancer) at the time of blood draw. All aspects of this study were approved by the Partners’ HealthCare System’s institutional review board. The participants’ return of a completed questionnaire was accepted as informed consent and was approved by the review board.

### Identification of rheumatoid arthritis cases

Methods of RA case identification and validation have been described in detail in past publications [22,23]. Briefly, nurses who self-reported a doctor-diagnosed connective tissue disease underwent a screening questionnaire for symptoms using the Connective Tissue Diseases Screening Questionnaire [24]. If the result was positive, a detailed medical record review was performed to determine definite RA using the American College of Rheumatology (ACR) classification criteria [25]. Individuals who met four of seven of the ACR criteria documented in the medical record were defined as cases. There were a small number of cases included as incident RA with only three of the ACR criteria and a physician’s diagnosis, but further agreed upon by two rheumatologists on the basis of chart review (*n* = 8). There were 11 cases with a missing date of symptom onset; in those cases, we therefore imputed the time of onset by assuming that the time between onset and diagnosis was similar to those in the rest of the cases (median time between onset and diagnosis was 6 months). Only cases with a blood draw at least 3 months before symptom onset were included.

### Identification of matched controls

Three controls for each confirmed incident RA case were randomly chosen from among participants with stored blood, matched on age (±1 year), menopausal status and postmenopausal hormone use, as well as on time of data collection and fasting status at blood draw. In NHSII, premenopausal women were also matched on timing of blood sample in the menstrual cycle. We excluded from the control group any self-reported RA not confirmed by a doctor and any participants who reported other connective tissue diseases.

### Laboratory methods

#### Anticitrullinated peptide antibodies

Potential RA-associated antigens identified from the literature and through proteomic screening [26] were coupled to spectrally distinct beads using the BioPlex multiplex assay platform (Bio-Rad Laboratories, Hercules, CA, USA) for analysis and a Luminex 200 instrument (Luminex, Austin, TX, USA) as previously described [6]. Three preestablished control serum samples consisting of high reactivity, low reactivity and no reactivity were run on each plate as internal controls: negative, low positive and high positive. Reactivities to 18 ACPAs targeted to synovial epitopes were measured in raw fluorescent intensity units and determined to be positive if they were more than three times the standard deviation above the mean in controls. A less conservative cutoff of more than two times the standard deviation was used in sensitivity analysis. ACPA targets included citrullinated enolase, biglycan, clusterin, fibrinogen, histone 2A, histone 2B and vimentin. Three native peptides were also assayed to serve as controls (fibrinogen, histone 2B and vimentin). A detailed list of each ACPA target can be found in Additional file 1: Table S1. Intrabatch coefficients of variation (CVs) were 4% to 10%, and interbatch CVs were 6% to 16%. We examined reactivity to any ACPA overall, ACPAs by peptide group (for example, reactivity to any citrullinated clusterin peptide) and individual ACPAs. A count of ACPAs was carried out by adding the number of positive ACPAs. An anti-CCP second-generation assay was performed by bead-based microarray using beads coated with peptide antigen (generously provided by Bio-Rad Laboratories). A relative fluorescence value greater than 1,500 was considered anti-CCP-positive, as this cutoff was determined to have similar sensitivity and specificity to a variety of plate-based, anti-CCP2 enzyme-linked immunosorbent assays.

#### HLA-SE

HLA low to intermediate typing was performed by One Lambda LABType reverse SSO Luminex DNA typing method (One Lambda, Canoga Park, CA, USA). First, target DNA was PCR-amplified using biotinylated group-specific primers. The PCR product was then denatured and hybridized to sequence-specific oligonucleotide probes bound to fluorescently coded microspheres to identify alleles encoded by the sample DNA. A flow analyzer, the Luminex 200, identified fluorescence intensity of R-phycoerythrin-conjugated streptavidin on each microsphere. The assignment of the HLA typing was based on the reaction pattern compared to probe specificities associated with published HLA gene sequences. HLA high-resolution typing was performed by sequencing. HLA locus-specific amplification was performed in a thermal cycler using the amplification primer mix and template DNA. Sequencing was performed using BigDye Terminator sequencing chemistry (Applied Biosystems, Foster City, CA, USA). Group-specific sequencing primers were used when necessary to resolve allele-specific ambiguities. We defined *HLA-SE*-positive as those having at least one SE allele (HLA-DRB*0401, 0404, 0405, 0408, 0101, 0102, 1001 or 09). *HLA-SE* data were available for 190 cases and 283 controls from another study. The distribution of demographics and potential confounders in this subset with *HLA-SE* data was similar to that of the full study population.

### Statistical analysis

Covariates were collected from the questionnaire before blood draw and were selected for analysis if associated with RA. Continuous variables included were pack-years of smoking, measured by the product of years of smoking and packs of cigarettes per day [27-29]; cumulative average alcohol intake in grams per day [28,30]; and body mass index (BMI) in kilograms per square meter [31]. Less than 1% of study participants had missing data for each continuous covariate; therefore, median values from the control group were imputed. Irregular menses [22] was included as a dichotomous variable, and an indicator was used for missing data.

Risk ratios (RRs) and their 95% confidence intervals (95% CIs) were obtained from conditional logistic regression models. Multivariable models included age at blood draw, pack-years of smoking, BMI, alcohol intake and irregular menses. Multivariable Cox proportional hazards models, including an interaction term for each ACPA and time from blood draw to symptom onset, were used to examine whether the association between each ACPA and RA varied over time. Preclinical RA cases were stratified into subgroups based on time between blood draw and onset of RA. Because each participant donated a single blood sample, the subgroups were mutually exclusive. Effect modification of the association between ACPA positivity and RA by *HLA-SE* was assessed using unconditional logistic regression with adjustment for matching factors. The ratio of odds ratios was calculated to examine the multiplicative interaction between ACPA and *HLA-SE*, and additive interaction was assessed by calculating the synergy index (S) [32]. A χ^2^ test for trend was used to compare categorical ACPA count over time intervals. To determine if *HLA-SE* was associated with peptide-specific ACPAs, we used logistic regression models.

## Results

We included 192 cases and 567 controls in the study (8 matched sets had fewer than 3 controls). On average, compared to controls, cases were more likely to have ever smoked cigarettes and drank less alcohol prior to blood draw (Table 1). Cases had a mean age of 59.9 years at diagnosis, and the median time between blood draw and diagnosis was 7.3 years (range: 4 months to 17.3 years). At the time of blood draw, 25.0% of cases and 6.7% of controls were positive for at least one ACPA (Table 1).

**Table 1:** **Characteristics of preclinical rheumatoid arthritis cases and matched controls at time of blood collection in the Nurses’ Health Study and Nurses’ Health Study II**^**a**^

**Characteristics**	**RA cases (*N*** **= 192)**	**Matched controls (*N*** **= 567)**
Cohort, *n* (%)		
NHS	136 (70.8)	405 (71.4)
NHSII	56 (29.2)	162 (28.6)
Caucasian, *n* (%)	190 (99.0)	556 (98.1)
Mean age at blood collection, years (SD)	52.3 (8.0)	52.4 (8.1)
Never smoker, *n* (%)	83 (43.2)	279 (49.2)
Current smoker, *n* (%)	33 (17.2)	82 (14.5)
Past smoker, *n* (%)	76 (39.6)	206 (36.3)
Mean pack-years of smoking^b^ (SD)	23.1 (16.4)	20.8 (18.8)
Mean alcohol intake, g/day (SD)	4.2 (5.6)	5.7 (8.9)
Mean BMI, kg/m^2^ (SD)	26.1 (5.0)	25.0 (4.4)
Premenopausal, *n* (%)	65 (33.9)	193 (34.0)
Parous, *n* (%)	175 (91.1)	511 (90.1)
Irregular menses, *n* (%)	32 (16.7)	56 (9.9)
Positive for any ACPA, *n* (%)	48 (25.0)	38 (6.7)
Positive for anti-CCP, *n* (%)	23 (12.0)	0 (0)
Mean age at RA diagnosis, years (SD)	59.9 (9.9)	
Mean time to RA onset, months (SD)	91.1 (53.6)	
Median time to RA onset, months (IQR)	87.5 (92.0)	
Morning stiffness, *n* (%)	143 (74.5)	
Arthritis three or more joint areas, *n* (%)	176 (91.7)	
Hand arthritis, *n* (%)	191 (99.5)	
Symmetric arthritis, *n* (%)	188 (97.9)	
Nodules, *n* (%)	21 (10.9)	
Erosions, *n* (%)	43 (22.4)	

^a^ACPA: anticitrullinated peptide autoantibody, BMI: body mass index, CCP: cyclic citrullinated peptide, NHS: Nurses’ Health Study, NHSII: Nurses’ Health Study II, RA: rheumatoid arthritis. ^b^Pack-years among ever smokers only.

The RR associated with any ACPA-positive was 4.9 (95% CI = 2.9 to 8.2; *P* < 0.0001). Each ACPA within each peptide type was significantly associated with risk of RA. RRs ranged from 4.7 to 11.7 (Table 2). All individual ACPAs were associated with RA, except for fibrinogen A 211–230 citrullinated cyclic (Additional file 1: Table S1).

**Table 2:** **Anticitrullinated peptide autoantibodies in women and risk of future rheumatoid arthritis in the Nurses’ Health Study and the Nurses’ Health Study II**^**a**^

**ACPA target**	**Positive cases (*N*** **= 192)**	**Positive controls (*N*** **= 567)**	**Unadjusted RR (95% CI)**	***P *** **value**	**Adjusted RR (95% CI)**	***P *** **value**
Any ACPA	48	38	4.9 (3.0 to 7.8)	<0.0001	4.9 (2.9 to 8.2)	<0.0001
Biglycan	10	5	6.0 (2.1 to 17.5)	0.001	4.7 (1.6 to 14.2)	0.006
Clusterin	25	7	12.2 (5.0 to 29.8)	<0.0001	11.7 (4.7 to 26.3)	<0.0001
Enolase	5	3	5.0 (1.2 to 20.9)	0.03	5.8 (1.3 to 27.1)	0.02
Fibrinogen	37	22	6.3 (3.5 to 11.4)	<0.0001	5.9 (3.2 to 11.0)	<0.0001
Histone 2A	13	8	4.9 (2.0 to 11.8)	<0.001	5.3 (2.1 to 13.2)	<0.001
Histone 2B	18	8	7.3 (3.1 to 17.6)	<0.0001	7.0 (2.8 to 17.2)	<0.0001
Vimentin	28	11	9.8 (4.5 to 21.6)	<0.0001	9.7 (4.3 to 21.8)	<0.0001

^a^ACPA: anticitrullinated peptide autoantibody, CI: confidence interval, RR, risk ratio. RR and 95% CI were estimated by using conditional logistic regression models including matching factors and further adjusted for age at blood draw, alcohol intake, body mass index, regularity of menses and pack-years of smoking.

### Relationship of anticitrullinated peptide autoantibodies to time to rheumatoid arthritis onset

**Figure 1:**
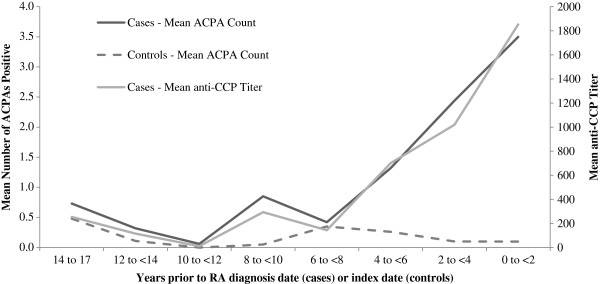
**Mean anticitrullinated peptide antibody count and anticyclic citrullinated peptide titer by years prior to rheumatoid arthritis diagnosis.** Mean number of positive anticitrullinated peptide antibodies (ACPAs) and anticyclic citrullinated peptide (anti-CCP) titer by time between blood draw and rheumatoid arthritis (RA) diagnosis date in cases and index date in controls in the Nurses’ Health Study (NHS) and the Nurses’ Health Study II (NHSII). ELISA: enzyme-linked immunosorbent assay.

The association between each ACPA and RA varied significantly by time from blood draw to onset (*P* < 0.0001 for interaction with time in Cox proportional hazards model). Therefore, we examined the associations among pre-RA subgroups stratified by time to onset within three time windows (less than 5 years, 5 years to less than 10 years and 10 years or longer). The highest reactivity for each ACPA was observed in the subgroup within the time interval closest to onset (Figure 1). Any ACPA-positive was associated with an almost 18-fold increased risk for the subgroup within 5 years of onset (RR = 17.6 (95% CI = 5.8 to 53.7)) and a 4-fold increased risk for the subgroup with blood draws 5 to 10 years before onset (RR = 4.2 (95% CI = 1.7 to 10.4)). There was no statistically significant increased risk in the subgroup with blood draws 10 years or more before RA onset (RR = 1.4 (95% CI = 0.5 to 4.3)) (Figure 1).

**Figure 2:**
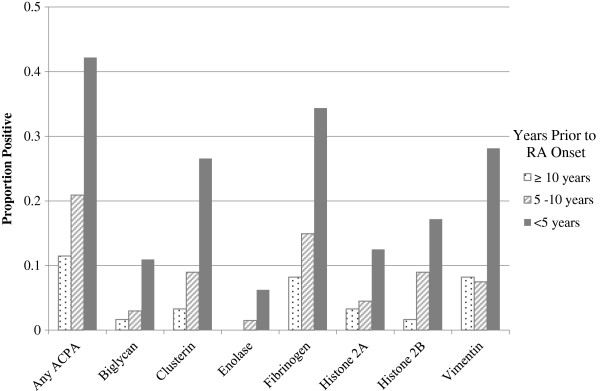
**Proportion anticitrullinated peptide antibody-positive cases in pre–rheumatoid arthritis cases by years prior to rheumatoid arthritis diagnosis.** Proportion of positive cases for any anticitrullinated peptide antibody (ACPA) and peptide-specific ACPAs in pre–rheumatoid arthritis (RA) cases with blood drawn 10 or more years (*n* = 61), 5 years to less than 10 years (*n* = 67) and less than 5 years before diagnosis (*n* = 64) in the Nurses’ Health Study and Nurses’ Health Study II. CI: confidence interval, RR: risk ratio.

**Figure 3:**
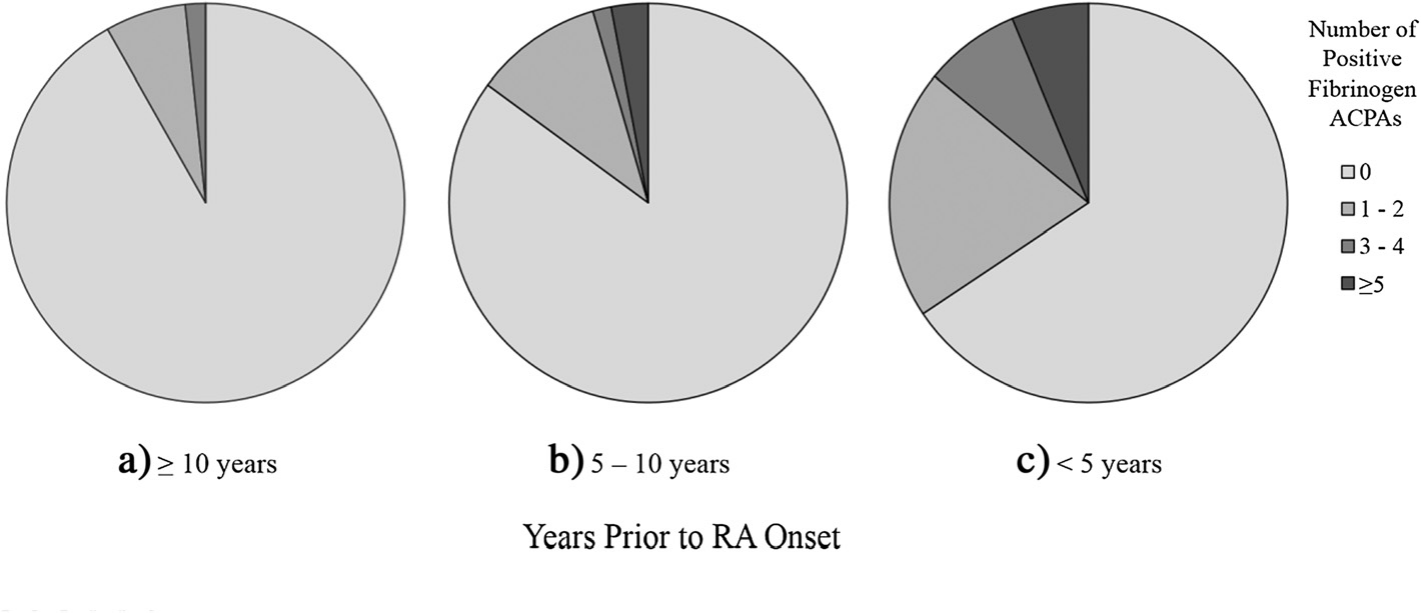
**Number of anticitrullinated fibrinogen antibodies in pre–rheumatoid arthritis cases by years prior to rheumatoid arthritis diagnosis.** Number of anticitrullinated fibrinogen antibodies in subgroups of cases diagnosed **(a)** 10 years or more **(b)** 5 years to less than 10 years and **(c)** less than 5 years before diagnosis of rheumatoid arthritis (RA) in the Nurses’ Health Study and Nurses’ Health Study II. ACPA: anticitrullinated peptide antibody.

Among cases, the number of positive ACPAs was higher the closer the blood draw was to onset. Cases with RA onset sooner after blood draw showed, on average, more reactivity to more ACPAs (Figure 2). We observed a similar trend in higher anti-CCP titers the closer to onset. The number of specific positive ACPAs by time period in cases and controls is listed in Additional file 2: Table S2. When we examined fibrinogen alone, for which we tested seven separate citrullinated peptide reactivities, we found that the number of citrullinated fibrinogen antibodies was higher in those cases with blood draw closer to onset (Figure 3) (*P* = 0.003 by χ^2^ test for trend).

When cases were restricted to those diagnosed within 5 years of blood draw, the sensitivity of an ACPA-positive test was 42.2% and the specificity was 93.3%. In comparison, anti-CCP-positive sensitivity was 26.6% and the specificity was 100%. Characteristics of the RA cases diagnosed within 5 years of blood draw are described in Additional file 3: Table S3.

### Relationship between anticitrullinated peptide autoantibody and HLA-SE

**Figure 4:**
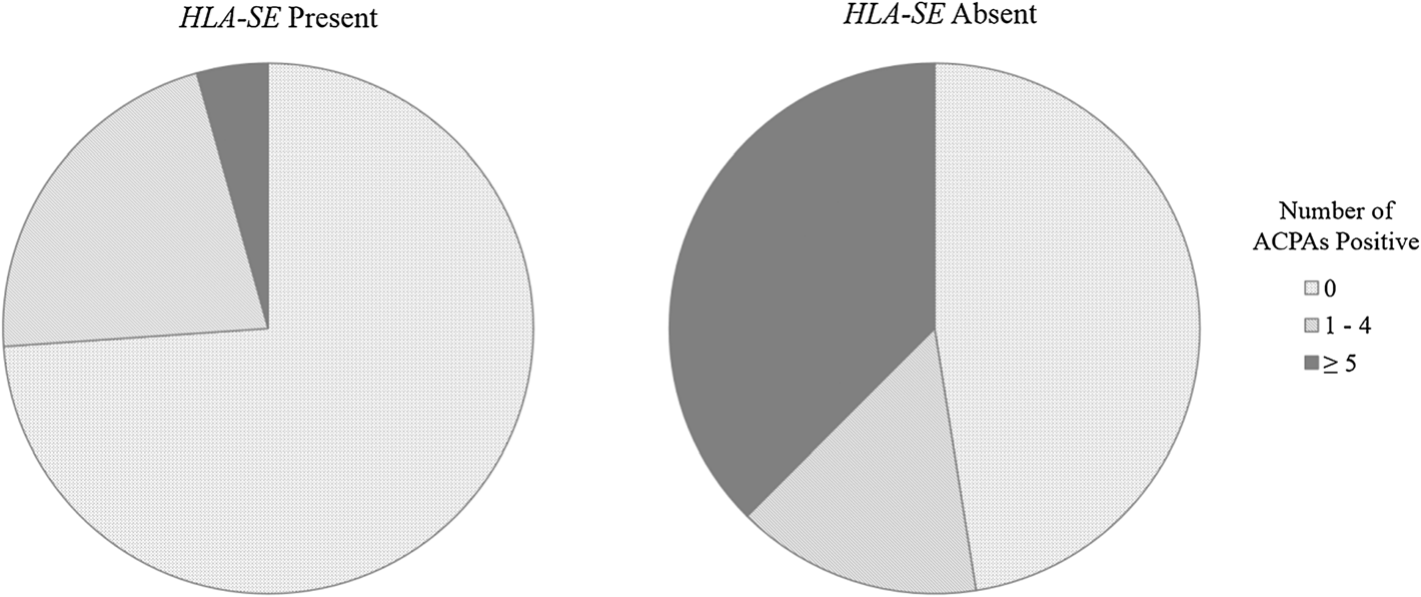
**Number of anticitrullinated peptide autoantibodies positive by HLA-SE presence in pre–rheumatoid arthritis cases less than 5 years before diagnosis.** Number of anticitrullinated peptide antibodies (ACPAs) positive in human leukocyte antigen shared epitope (*HLA-SE*)-negative (*n* = 23) and *HLA-SE*-positive (*n* = 40) cases diagnosed within 5 years of blood draw in the Nurses’ Health Study and Nurses’ Health Study II. *P* = 0.01 by χ^2^ test for trend.

The *HLA-SE*-positive/ACPA-positive group carried the highest risk of RA. Comparing those who were *HLA-SE*-positive and ACPA-positive to those who were negative for both, the overall OR was 10.4 (95% CI = 4.3 to 25.2). When we compared just those with blood draws less than 5 years before RA onset, the OR was 33.3 (95% CI = 11.1 to 99.6) (Table 3). There was a positive but not statistically significant interaction for the additive scale, suggesting the presence of excess risk from interaction between ACPA and *HLA-SE* relative to the risk from ACPA and *HLA-SE* without interaction (S = 4.5 (95% CI = 0.9 to 24.5); *P* = 0.06). RA patients who were *HLA-SE*-positive showed reactivity to more ACPA types than those who were *HLA-SE*-negative (*P* = 0.01 by χ^2^ test for trend) (Figure 4). Only 1 of 23 *HLA-SE*-negative cases had five or more positive ACPAs, whereas 15 of 40 *HLA-SE*-positive cases were positive for five or more ACPAs. We further examined the association between *HLA-SE* and each ACPA using logistic regression models for cases only. We found that antibodies to citrullinated clusterin and vimentin were associated with *HLA-SE* in patients diagnosed within any time period after blood draw. *HLA-SE* was associated with citrullinated fibrinogen only within the time interval closest to onset (*P* < 0.05).

**Table 3:** **Odds ratios for rheumatoid arthritis by anticitrullinated peptide autoantibody positivity and presence of *HLA-SE***^**a**^

	**ACPA-negative**			**ACPA-positive**			
**Parameter**	**Cases (*N*** **= 36)**	**Controls (*N*** **= 268)**	**OR (95% CI)**	**Cases (*N*** **= 27)**	**Controls (*N*** **= 15)**	**OR (95% CI)**	**OR (95% CI) for ACPA within strata of *HLA-SE***
*HLA-SE* absent	17	153	1.0 (ref)	6	8	7.7 (2.2 to 27.5)	7.7 (2.2 to 27.5)
						*P* = 0.002	*P* = 0.002
*HLA-SE* present	19	115	1.4 (0.7 to 2.9)	21	7	33.3 (11.1 to 99.6)	23.8 (7.9 to 71.8)
			*P* = 0.38			*P* < 0.0001	*P* < 0.0001
OR (95% CI) for *HLA-SE* present			1.4 (0.7 to 2.9)			4.3 (1.0 to 18.6)	
within strata of ACPA			*P* = 0.38			*P* = 0.05	

^a^ACPA: anticitrullinated antibody, CI: confidence interval, HLA-SE: human leukocyte antigen shared epitope, OR, odds ratio, ref: reference value. Measure of effect modification on the additive scale: Synergy Index = 4.5 (95% CI = 0.9 to 21.6; *P* = 0.06). Measure of effect modification on the multiplicative scale: ratio of odds ratios = 3.1 (95% CI = 0.6 to 15.8; *P* = 0.18). ORs estimated by logistic regression models adjusted for matching factors and age at blood draw, alcohol intake, body mass index, regularity of menses and pack-years of smoking. Restricted to cases with blood draw less than 5 years before diagnosis (cases = 63, controls = 283) in the Nurses’ Health Study and Nurses’ Health Study II.

## Discussion

In this large, prospective cohort study of women followed for many years, we have demonstrated that the majority of a panel of 18 different ACPAs targeted to epitopes in the rheumatoid synovium are strongly associated with risk of future RA. The highest reactivity for each of these ACPAs was observed in the subgroup within 5 years of RA symptom onset. Having any ACPA reactivity was associated with an almost 18-fold increased risk of RA among the subgroup within 5 years of RA symptom onset and a 4-fold increased risk within the subgroup with blood draws 5 to 10 years before RA symptom onset. The higher number of ACPAs and the higher overall ACPA reactivity closer to the time of RA onset may be indicative of epitope spreading, as has been reported in prior studies [5,6,9,10]. We also found that *HLA-SE*-positive RA cases showed reactivity to more ACPA subtypes than *HLA-SE*-negative cases and that antibodies to citrullinated clusterin and vimentin were most strongly associated with *HLA-SE*-positive RA.

Three recent case–control studies have identified individuals with stored blood who later developed RA. A blood donor study in the Netherlands examined five ACPAs in seventy-nine donors who gave blood multiple times before they developed RA [5]. Another study analyzed 17 ACPAs using a multiplex panel similar to the panels used in our study in stored serum samples from the US Department of Defense Serum Repository, including 81 individuals who had blood samples taken before RA diagnosis [6]. A third study examined 10 ACPAs in 374 individuals who had donated blood in Sweden before RA diagnosis [10]. (A subset of these cases was reported by van der Woude *et al*. [9]). These pre-RA studies also showed increased ACPA reactivity in time periods closest to disease diagnosis and an expansion of the ACPA repertoire over time among cases. Our results are not directly comparable to those from the Netherlands and Sweden, as we used a different panel of ACPAs. However, we employed the same panel of ACPAs tested in the prior US study [6] and found that all peptide-specific ACPAs were associated with a fivefold or greater increased risk of RA.

None of the blood bank studies cited above examined the combined effect of ACPA positivity and *HLA-SE* on RA risk, however, nor were the investigators able to adjust for potential confounders such as smoking. In the current study, the presence of *HLA-SE* and any ACPA together conferred an increased risk of RA: a 10-fold increased risk overall and a 33-fold increased risk within 5 years of RA onset. This particular group of individuals is at an especially high risk of imminent RA.

One previous case–control study of pre-RA cases from a Swedish blood donor bank compared those with *HLA-SE* alleles and commercial anti-CCP assay positivity to those with neither and calculated an OR for RA of 66.8 (95% CI = 8.3 to 549.4) [15]. Researchers in previous studies that included patients with established RA or early RA symptoms have found that the combination of a positive anti-CCP test measured close to the time of diagnosis of RA or individual ACPAs and *HLA-SE* was greater than the effect of each alone [14,16,33]. However, these findings address a different question: whether ACPAs and *HLA-SE* are predictive of RA among patients who already have symptoms.

Among the NHS participants who later developed RA, those with *HLA-SE* had a more diverse ACPA repertoire. Hill and colleagues have demonstrated that major histocompatibility complex class II molecules containing the shared epitope had higher affinity for citrullinated peptides in mice and that citrullinated peptide modification activated a CD4+ T-cell response [34]. We observed that *HLA-SE* was associated with anticitrullinated vimentin and fibrinogen reactivity in particular, which is congruent with reports by van der Woude *et al*. (vimentin) [9] and van de Stadt *et al*. (fibrinogen) [8]. We have further demonstrated that *HLA-SE* was associated with antibodies to citrullinated clusterin. In this study, we have included ACPAs directed toward well-established peptide targets (fibrinogen, vimentin and enolase [35]) as well as newer targets (biglycan, clusterin and histones 2A and 2B), all of which have been shown to be associated with RA. The association between 17 different citrullinated peptides (7 different peptide types) and RA risk suggests that the magnitude and diversity of recognition to many different ACPAs is key to understanding the immune response in the preclinical time period [36].

The generalizability of our study may be limited, as we studied the NHS cohorts, which include only women, but it is thought that RA pathogenesis is similar in men and women. We had only one blood sample per individual; therefore, it was not possible to investigate epitope spreading over time in a single individual. With a follow-up after blood draw of up to 17 years (longer follow-up than previous studies of pre-RA), we were able to investigate if there was an early rise in ACPAs more than 10 years prior to RA onset. Our study helps to increase understanding of the diversity of ACPAs before onset using a relatively large sample of pre-RA cases. We included a large, up-to-date ACPA panel, which enabled us to study ACPA count in relation to *HLA-SE* and RA onset date, which may be more relevant than one ACPA alone in the years leading up to disease onset. This study is also strengthened by the use of data on health and lifestyle factors taken from questionnaires before blood draw, which allowed us to adjust for potential confounders.

## Conclusions

We found that a diverse ACPA repertoire is associated with *HLA-SE* and is strongest within 5 years of onset of RA. Our study suggests that there is a broader citrullinated epitope recognition profile instead of one ACPA being responsible for inciting disease. The magnitude of the response to ACPAs in combination with the presence of *HLA-SE* may be most important for determining the risk of disease. In the future, researchers should continue to investigate whether individual environmental factors trigger the development of ACPA reactivity and immune dysregulation in the peripheral blood before RA onset.

## Abbreviations

ACPA: Anticitrullinated peptide autoantibody; ACR: American College of Rheumatology; anti-CCP: Anticyclic citrullinated peptide; BMI: Body mass index; CI: Confidence interval; HLA-SE: Human leukocyte antigen shared epitope; NHS: Nurses’ Health Study; OR: Odds ratio; RA: Rheumatoid arthritis; ROR: Ratio of odds ratio; RR: Risk ratio; S: Synergy index.

## Competing interests

The authors declare that they have no competing interests.

## Authors’ contributions

EVA, BLG, SM, BR, FG, EWK and KHC contributed to the study conception and design. SM helped with data management. EVA performed the analysis. EVA, BR, FG, EWK and KHC interpreted the results and drafted the paper. JS, WR and CAW performed the laboratory analysis. All authors contributed to editing the draft for content and approved the final version of the paper.

## Supplementary Material

Additional file 1: Table S1 Anti-Citrullinated Peptide Autoantibodies in Women and Risk of Future Rheumatoid Arthritis in NHS and NHSII. Click here for file

Additional file 2: Table S2 Number of positive anti-citrullinated peptide autoantibodies in preclinical RA cases and their matched controls by time prior to RA onset (< 5 years, 5–10 years, ≥10 years) NHS and NHSII. Click here for file

Additional file 3: Table S3 Characteristics of preclinical RA cases with blood drawn less than 5 years before diagnosis (N = 64) in NHS and NHSII. Click here for file
