# Supplementary material for: Anti-citrullinated peptide autoantibodies, human leukocyte antigen shared epitope and risk of future rheumatoid arthritis: a nested case–control study
Source: Arthritis Res Ther. 2013 Oct 23;15(5):R159. doi: 10.1186/ar4342 (PMC3953952; doi:10.1186/ar4342)
Supplement: Additional file 1: Table S1 — Anti-Citrullinated Peptide Autoantibodies in Women and Risk of Future Rheumatoid Arthritis in NHS and NHSII. [file ar4342-S1.doc]

**Additional File1**

**Table S1**. Anti-Citrullinated Peptide Autoantibodies in Women and Risk of Future Rheumatoid Arthritis in NHS and NHSII.

| **ACPA** | **No. Positive Cases**  **(Total N=192)** | **No. Positive**  **Controls**  **(Total N=567)** | **Adjusted RR*(95% CI)** | **p value** |
| --- | --- | --- | --- | --- |
| Biglycan 247-266 Cit Cyclic | 10 | 5 | 4.7 (1.6, 14.1) | 0.006 |
| Clusterin 221-240 Cit Cyclic | 13 | 5 | 8.5 (3.0, 22.8) | <0.0001 |
| Clusterin 231-250 Cit cyclic | 21 | 6 | 11.5 (4.2, 31.6) | <0.0001 |
| Clusterin 231-250 Cit | 22 | 5 | 13.9 (4.7, 41.4) | <0.0001 |
| Enolase 1A 5-21 Cit | 5 | 3 | 5.8 (1.3, 27.1) | 0.02 |
| Fibrinogen Cit | 10 | 4 | 6.6 (2.0, 21.9) | 0.002 |
| Fibrinogen A 41-60 Cit3 Cyclic | 15 | 9 | 4.8 (2.0, 11.5) | <0.001 |
| Fibrinogen A 211-230 Cit Cyclic | 3 | 7 | 1.4 (0.3, 5.3) | 0.66 |
| Fibrinogen A 556-575 Cit | 7 | 4 | 4.3 (1.2, 15.3) | 0.03 |
| Fibrinogen A 556-575 Cit Cycilc | 19 | 5 | 10.2 (3.7, 28.2) | <0.0001 |
| Fibrinogen A 616-635 Cit3 | 14 | 6 | 7.2 (2.5, 20.8) | <0.001 |
| Fibrinogen A 616-635 Cit3 Cyclic | 24 | 6 | 17.6 (5.9, 52.5) | <0.0001 |
| Histone 2A 1-20 Cit | 8 | 6 | 3.9 (1.3, 11.6) | 0.01 |
| Histone 2A 1-20 Cit Cyclic | 9 | 4 | 7.7 (2.3, 26.3) | 0.001 |
| Histone 2B 62-81 Cit Cyclic | 7 | 4 | 5.2 (1.5, 18.4) | 0.01 |
| H2B Cit | 13 | 4 | 8.3 (2.6, 26.2) | <0.001 |
| Vimentin Cit | 17 | 6 | 11.1 (4.0, 31.0) | <0.0001 |
| Vimentin 58-77 Cit3 Cyclic | 21 | 5 | 11.9 (4.4, 32.5) | <0.0001 |
| Anti-CCP | 23 | 0 | - | - |
| 2 or more positive ACPAs | 35 | 13 | 11.1 (5.2, 23.7) | <0.0001 |

*Risk ratio (RR) and 95% confidence interval (95%CI) estimated by conditional logistic regression models including matching factors and further adjusted for age at blood draw, alcohol intake, body mass index, regularity of menses and pack-years smoking.
