# Supplementary material for: Anti-citrullinated peptide autoantibodies, human leukocyte antigen shared epitope and risk of future rheumatoid arthritis: a nested case–control study
Source: Arthritis Res Ther. 2013 Oct 23;15(5):R159. doi: 10.1186/ar4342 (PMC3953952; doi:10.1186/ar4342)
Supplement: Additional file 2: Table S2 — Number of positive anti-citrullinated peptide autoantibodies in preclinical RA cases and their matched controls by time prior to RA onset (< 5 years, 5–10 years, ≥10 years) NHS and NHSII. [file ar4342-S2.doc]

**Additional File2**

**Table S2.** Number of Anti-citrullinated peptide autoantibodies positive in preclinical RA cases and their matched controls by time prior to RA onset (< 5 years, 5-10 years, ≥10 years) NHS and NHSII.

|  | **< 5 years** | | **5 – 10 years** | | **≥ 10 years** | |
| --- | --- | --- | --- | --- | --- | --- |
| **ACPA** | **Cases**  **(N=64)** | **Controls**  **(N=191)** | **Cases**  **(N=67)** | **Controls**  **(N=198)** | **Cases**  **(N=61)** | **Controls**  **(N=178)** |
| Biglycan 247-266 Cit Cyclic | 7 | 2 | 2 | 2 | 1 | 1 |
| Clusterin 221-240 Cit Cyclic | 8 | 1 | 3 | 2 | 2 | 2 |
| Clusterin 231-250 Cit cyclic | 15 | 1 | 5 | 1 | 1 | 4 |
| Clusterin 231-250 Cit | 16 | 1 | 5 | 1 | 1 | 3 |
| Enolase 1A 5-21 Cit | 4 | 2 | 1 | 1 | 0 | 0 |
| Fibrinogen Cit | 8 | 2 | 0 | 1 | 2 | 1 |
| Fibrinogen A 41-60 Cit3 Cyclic | 8 | 3 | 6 | 4 | 1 | 2 |
| Fibrinogen A 211-230 Cit Cyclic | 3 | 2 | 0 | 2 | 0 | 3 |
| Fibrinogen A 556-575 Cit | 4 | 1 | 3 | 1 | 0 | 2 |
| Fibrinogen A 556-575 Cit Cycilc | 13 | 1 | 4 | 2 | 2 | 2 |
| Fibrinogen A 616-635 Cit3 | 8 | 2 | 5 | 2 | 1 | 2 |
| Fibrinogen A 616-635 Cit3 Cyclic | 15 | 3 | 7 | 2 | 2 | 1 |
| Histone 2A 1-20 Cit | 4 | 1 | 2 | 3 | 2 | 2 |
| Histone 2A 1-20 Cit Cyclic | 7 | 1 | 2 | 2 | 0 | 1 |
| Histone 2B 62-81 Cit Cyclic | 4 | 1 | 2 | 2 | 1 | 1 |
| H2B Cit | 9 | 2 | 4 | 2 | 0 | 0 |
| Vimentin Cit | 10 | 4 | 4 | 0 | 3 | 2 |
| Vimentin 58-77 Cit3 Cyclic | 16 | 1 | 3 | 2 | 2 | 2 |
| Anti-CCP | 17 | 0 | 4 | 0 | 2 | 0 |
