# Supplementary material for: Anti-citrullinated peptide autoantibodies, human leukocyte antigen shared epitope and risk of future rheumatoid arthritis: a nested case–control study
Source: Arthritis Res Ther. 2013 Oct 23;15(5):R159. doi: 10.1186/ar4342 (PMC3953952; doi:10.1186/ar4342)
Supplement: Additional file 3: Table S3 — Characteristics of preclinical RA cases with blood drawn less than 5 years before diagnosis (N = 64) in NHS and NHSII. [file ar4342-S3.doc]

**Additional File3**

**Table S3.** Characteristics of preclinical RA cases with blood drawn less than 5 years before diagnosis (N=64) in NHS and NHSII.

|  | **RA Cases**  (N=64) |
| --- | --- |
| Cohort, N (%) |  |
| NHS | 35 |
| NHSII | 29 |
| Caucasian, N (%) | 64 (100) |
| Age at blood collection, years, mean (SD) | 50.5 (7.8) |
| Never smoker, N (%) | 32 (50.0) |
| Current Smoker, N (%) | 9 (14.1) |
| Past Smoker, N (%) | 23 (35.9) |
| Pack-years smoking*, mean (SD) | 20.9 (13.2) |
| Alcohol intake, grams/day, mean (SD) | 3.7 (4.8) |
| Body Mass Index, mean (SD) | 26.9 (5.8) |
| Premenopausal, N (%) | 24 (37.5) |
| Parous, N (%) | 59 (92.2) |
| Irregular menses, N (%) | 14 (21.9) |
| Positive for any ACPA, N (%) | 27 (42.2) |
| Positive for anti-CCP, N (%) | 17 (26.6) |
| Age at RA diagnosis, mean (SD) | 53.1 (7.9) |
| Time to RA onset, months, mean (SD) | 32.4 (15.9) |
| median (IQR) | 31.0 (27.0) |
| AM stiffness, N (%) | 45 (70.3) |
| Arthritis ≥3 joint areas, N (%) | 58 (90.6) |
| Hand Arthritis, N (%) | 64 (100) |
| Symmetric arthritis, N (%) | 64 (100) |
| Nodules, N (%) | 7 (10.9) |
| Erosions, N (%) | 18 (28.1) |

*pack-years among ever-smokers only

NHS; Nurses’ Health Study, NHSII; Nurses’ Health Study II, ACPA; anti-citrullinated peptide autoantibodies, anti-CCP; anti-cyclic citrullinated peptide
